# The structure of erastin-bound xCT–4F2hc complex reveals molecular mechanisms underlying erastin-induced ferroptosis

**DOI:** 10.1038/s41422-022-00642-w

**Published:** 2022-03-29

**Authors:** Renhong Yan, Enjun Xie, Yaning Li, Jin Li, Yuanyuan Zhang, Ximin Chi, Xueping Hu, Lei Xu, Tingjun Hou, Brent R. Stockwell, Junxia Min, Qiang Zhou, Fudi Wang

**Affiliations:** 1 Westlake Laboratory of Life Sciences and Biomedicine, Key Laboratory of Structural Biology of Zhejiang Province, Institute of Biology, Westlake Institute for Advanced Study, School of Life Sciences, Westlake University, Hangzhou, Zhejiang China; 2 The First Affiliated Hospital, The Fourth Affiliated Hospital, School of Public Health, Institute of Translational Medicine, Cancer Center, State Key Laboratory of Experimental Hematology, Zhejiang University School of Medicine, Hangzhou, Zhejiang China; 3 The First Affiliated Hospital, The Second Affiliated Hospital, Basic Medical Sciences, School of Public Health, Hengyang Medical School, University of South China, Hengyang, Hunan China; 4 Beijing Advanced Innovation Center for Structural Biology, Tsinghua-Peking Joint Center for Life Sciences, School of Life Sciences, Tsinghua University, Beijing, China; 5 Hangzhou Institute of Innovative Medicine, College of Pharmaceutical Sciences, Zhejiang University, Hangzhou, Zhejiang China; 6 Institute of Bioinformatics and Medical Engineering, Jiangsu University of Technology, Changzhou, Jiangsu China; 7 Department of Biological Sciences and Department of Chemistry, Columbia University, New York, NY USA; 8 Present Address: Department of Biochemistry, School of Medicine, Southern University of Science and Technology, Shenzhen, Guangdong China

**Keywords:** Cryoelectron microscopy, Cell death

Dear Editor,

Ferroptosis is an iron-dependent, non-apoptotic form of regulated cell death characterized by an accumulation of lipid- derived reactive oxygen species (ROS). The small-molecule compound erastin induces ferroptosis via inhibiting the cystine-glutamate antiporter system x_c_^–^, which consists of two subunits, namely the light chain xCT and the heavy chain 4F2hc (encoded by the *SLC7A11* and *SLC3A2* genes, respectively).^1–4^ Recent studies have shown that xCT (SLC7A11) regulates ferroptosis in liver fibrosis, cardiomyopathy, and numerous other pathophysiological processes.^5–8^ The complex formed by xCT and 4F2hc has also been suggested as a possible therapeutic target for cancer, as it is overexpressed in a wide variety of cancer types; moreover, inhibiting xCT impairs cystine uptake, causing an accumulation of ROS and suppressing tumor growth.^9,10^ However, the underlying molecular mechanisms remain unknown. Here, we overexpressed and then co-purified human xCT and 4F2hc, finding that they form a stable complex (Fig. 1a). To test whether this purified complex is functional, we then reconstituted the complex into liposomes and performed a counter-flow assay. We found that the wild-type (WT) xCT–4F2hc complex mediates the exchange of cystine and glutamate — measured as the uptake of ^14^C-labeled cystine — and this activity was significantly reduced by the system x_c_^–^ inhibitors, erastin and sulfasalazine (Supplementary information, Fig. S1).

**Fig. 1:**
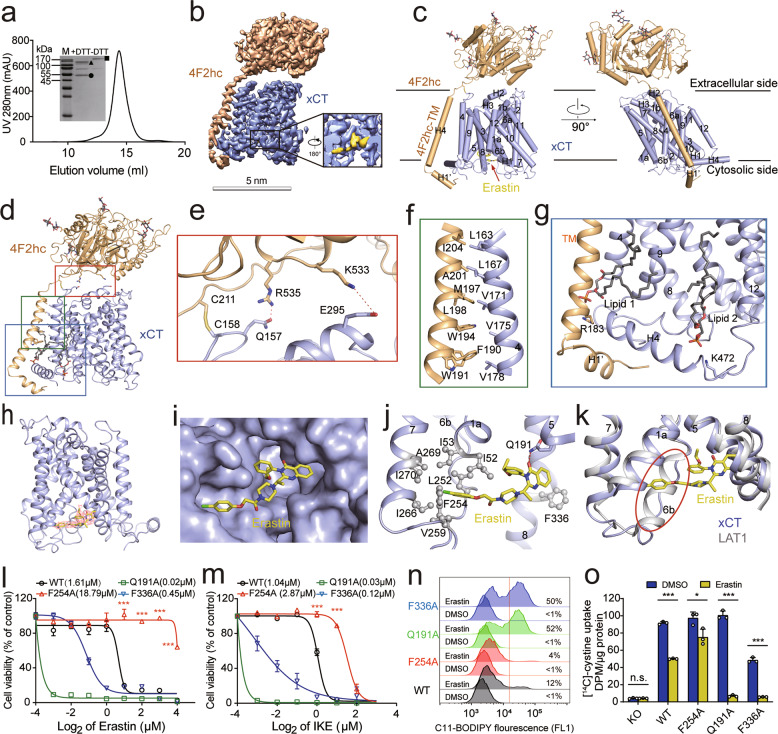
Cryo-EM structure of the erastin-bound xCT–4F2hc complex and the inhibitory effect of erastin on xCT activity in ferroptosis. **a** Representative gel filtration purification of the xCT–4F2hc complex. The inset shows a Coomassie blue-stained SDS-PAGE gel under reducing (+DTT) and oxidizing (–DTT) conditions. M, molecular weight marker. The bands corresponding to 4F2hc, xCT, and the disulfide-linked xCT–4F2hc complex are labeled with a triangle, circle, and square, respectively. **b**, **c** Cryo-EM map (**b**) and atomic model (**c**) of the erastin-bound xCT–4F2hc complex. **d** Interaction interfaces between xCT and 4F2hc. The boxed regions (from top to bottom) are shown in **e**, **f** and **g**. **e** Detailed view of the interactions at the extracellular interface, with the polar interactions shown as red dashed lines. **f** Detailed view of the hydrophobic interactions between the transmembrane helix in 4F2hc and TM4 in xCT. **g** Detailed view of the xCT–4F2hc complex at the intracellular side; xCT is bound to two lipid molecules. **h** Structure of the erastin-bound xCT subunit, with the density for erastin shown in mesh. **i** Model of the erastin-binding pocket located in the intracellular vestibule of xCT. **j** The erastin molecule is shown sandwiched between TM domains in xCT; the tail of the erastin molecule is surrounded by a cluster of hydrophobic residues (shown as spheres). **k** Overlay showing the structure of the TM domains in erastin-bound xCT and erastin-bound LAT1 (PDB ID: 6IRT). **l**, **m** Dose-response curves for erastin (**l**) and IKE (**m**) measured in xCT-knockout HT-1080 cells expressing either WT xCT or the indicated xCT mutants; the corresponding IC_50_ values are also shown. **n** Erastin-induced lipid peroxidation was measured using C11-BODIPY in xCT-knockout HT-1080 cells expressing either WT xCT or the indicated xCT mutants, 12 h after treatment with 2 μM erastin or DMSO as a negative control. The corresponding percentage of C11-BODIPY-positive values are shown. **o** Na^+^-independent [^14^C]-cystine uptake was measured in xCT-knockout HT-1080 cells and in cells expressing either WT xCT or the indicated xCT mutants; where indicated, the cells were pretreated for 2 h with either 4 μM erastin or DMSO. In **l**, **m** ***P < 0.001 vs WT (one-way ANOVA); in **o**, *P < 0.05, ***P < 0.001, and n.s., not significant (*P* > 0.05) (Student’s *t*-test).

Several additional inhibitors of xCT have also been identified, including the erastin analogs imidazole ketone erastin (IKE) and piperazine erastin (PE), as well as sorafenib and HG106. Among these inhibitors, erastin is commonly used to induce ferroptosis, particularly in cultured cells. Erastin functions by inhibiting the import of cystine, thereby depleting intracellular glutathione (GSH), which serves as a necessary cofactor for the enzyme glutathione peroxidase 4 (GPX4) in eliminating lipid peroxides.^1^ Despite its potential for cancer therapy, however, erastin has not reached the clinic due to its low water solubility and unstable metabolism when administered in vivo. Therefore, obtaining detailed information regarding the interaction between the xCT–4F2hc complex and erastin will likely provide new mechanistic and structural insights, facilitating the development of more effective ferroptosis-inducing therapies designed to target cancer cells.

Here, we used cryogenic electron microscopy (cryo-EM) to determine the structure of the erastin-bound xCT–4F2hc complex at 3.4 Å resolution (Fig. 1b, c; Supplementary information, Figs. S2–S4 and Table S1). Using this approach, nearly all of the sequences in both the xCT and 4F2hc subunits were resolved, with the exceptions of N-terminal residues 1–45 in xCT and N-terminal residues 1–163 in 4F2hc, which were not visible in the cryo-EM map. The overall structure of the xCT–4F2hc complex (Fig. 1d) is similar to that of the previously reported complex between the l-type amino acid transporter 1 (LAT1) and 4F2hc^11^ (Supplementary information, Fig. S5), with xCT in an inward-facing conformation. 4F2hc interacts with xCT at the extracellular interface and in the transmembrane region mainly through polar and hydrophobic interactions, respectively (Fig. 1e, f). The Cys158 residue in xCT forms a disulfide bond with the Cys211 residue in 4F2hc (Fig. 1e), and two lipid molecules bind to xCT at the transmembrane region near the intracellular side (Fig. 1g). Finally, the residues in 4F2hc that precede the transmembrane (TM) helix form a short helix (H1′) that fixes xCT at the intracellular side (Fig. 1g).

We also observed a well-resolved non-protein density in the intracellular vestibule of xCT, into which we incorporated an erastin molecule in the model, as the shape of this density is consistent with erastin (Fig. 1h). This erastin molecule fit well into the intracellular vestibule and was sandwiched between the TM domains of the xCT subunit (Fig. 1i, j). Specifically, the chlorophenoxy group at one end of the erastin molecule projects into a hydrophobic pocket lined by TM1a, TM6b, and TM7, with the benzene ring interacting with the Phe254 residue in TM6b (Fig. 1j). At the other end of the erastin molecule, the quinazolinol group is positioned in the hydrophobic pocket enclosed by TM5 and TM8, interacting with Gln191 and Phe336 (Fig. 1j). Interestingly, the secondary structure of TM6b, which forms a helix in LAT1,^11^ becomes a loop in xCT (Fig. 1k). Based on the structure of erastin-bound xCT, we then performed molecular docking using IKE, a more potent inhibitor of xCT than erastin.^12^ We found that IKE docked in the same pocket in xCT as erastin; in addition, IKE occupied a new sub-pocket formed by the residues Tyr251, Ala247, Lys198, and Ser330 (Supplementary information, Fig. S6).

To investigate the functional role of the xCT residues Gln191, Phe254 and Phe336 in mediating the interaction between xCT and erastin, we generated three mutant forms of Flag-tagged xCT (Q191A, F254A, and F336A) and expressed the WT and mutant xCT proteins in either HT-1080 cells (a human fibrosarcoma cell line) or HT-1080 cells lacking xCT (Supplementary information, Fig. S7a). We found that overexpressing F254A xCT made cells more resistant to both erastin- and IKE-induced ferroptosis than WT xCT, while overexpressing either the Q191A or F336A xCT were more sensitive to either erastin- or IKE-induced ferroptosis than WT xCT (Fig. 1l, m; Supplementary information, Fig. S7b, c). Notably, however, we found no significant difference between xCT-knockout cells expressing WT xCT and xCT-knockout cells expressing any of the xCT mutants when the cells were either cultured in cystine-free medium or treated with sulfasalazine (Supplementary information, Fig. S7d, e).

Given that ferroptosis is characterized by increased levels of ROS-induced lipid peroxidation,^1^ we used C11-BODIPY staining to measured lipid peroxidation in HT-1080 cells expressing an empty vector, WT xCT, or the mutant forms of xCT. Consistent with our cell viability results, we found that cells expressing either WT xCT or the F254A xCT mutant had significantly reduced levels of erastin-induced lipid peroxidation compared to cells expressing either the empty vector or the Q191A or F336A xCT mutant (Supplementary information, Fig. S7f, g). In all cases, co-treating the cells with the ferroptosis inhibitor ferrostatin-1 (Fer-1) abolished the erastin-induced increase in lipid peroxidation (Supplementary information, Fig. S7f, g). In xCT knockout HT-1080 cells, expressing the F254A xCT mutant rendered the cells more resistant to erastin-induced lipid peroxidation compared with WT xCT (Fig. 1n). Finally, similar results were obtained when we measured erastin-induced cell death, lipid peroxidation, and GSH/GSSG in HeLa cells expressing either WT xCT or the mutant forms of xCT (Supplementary information, Fig. S8a–e).

To further investigate whether these mutations in xCT affect the ability of erastin to inhibit the complex’s substrate transport activity, we performed a [^14^C]-cystine uptake assay using xCT-knockout HT-1080 cells expressing either WT xCT or mutant xCT. We found that cells expressing the F254A mutant were less sensitive to erastin-induced inhibition of cystine uptake (Fig. 1o; Supplementary information, Fig. S9). However, we found no significant difference between cells expressing the F254A mutant and WT xCT when the cells were treated with a high dose erastin (40 μM), which blocks ~90% of cystine uptake activity of xCT (Supplementary information, Fig. S9). To further characterize the functional consequences of the xCT mutations, we attempted to measure erastin–xCT binding using mass spectrometry. However, the results were inconsistent, possibly due to the poor water solubility of erastin and technical difficulties associated with attempting to quantify the binding affinity between a substrate and its binding transporter. For example, previous attempts using biophysical methods to measure binding between glucose and the glucose transporters GLUT1 and GLUT3 were unsuccessful, even though the structure of the glucose–GLUT3 complex was solved.^13^ Nevertheless, we acknowledge that the lack of erastin–xCT binding data is a limitation of this study. Based on our indirect functional data, however, we conclude that the F254A mutation only moderately affects the ability of erastin to inhibit xCT; indeed, it is possible that several mutations are needed to fully abolish erastin–xCT binding. Interestingly, we also found that both the Q191A and F336A xCT mutants were more sensitive to erastin than WT xCT (Fig. 1l, m); moreover, we found that the F336A mutant — but not the Q191A mutant — significantly affects the basal cystine transport activity of xCT, whereas both mutants increased the ability of erastin to inhibit cystine uptake (Fig. 1o). The precise mechanism underlying this effect remains unclear and warrants further study.

The recent studies reported the structures of the human xCT–4F2hc complex harboring consensus mutations in xCT at 6.2 Å resolution,^14^ and in apo state at 3.4 Å resolution or in complex with glutamate at 3.7 Å resolution, respectively, which provide a structural basis for understanding xCT substrate (glutamate and cystine) recognition.^15^ In contrast, we solved the cryo-EM structure of the erastin-bound human xCT–4F2hc complex at 3.4 Å resolution, revealing the site at which erastin binds and mediates the induction of ferroptosis. In this complex, the chlorophenoxy group in the erastin molecule functionally interacts with the Phe254 residue in the TM6b domain of xCT, thereby mediating erastin’s inhibitory effects of erastin and regulating erastin-induced ferroptosis; consistent with the finding that the chlorophenoxy moiety is required for inducting ferroptosis. Interestingly, we also found that the erastin analog IKE is a more potent inducer of ferroptosis than erastin, and a new sub-pocket formed by the residues Tyr251, Ala247, Lys198 and Ser330 residues in xCT may partially account for the higher potency of IKE (Supplementary information, Fig. S10a, b). Taken together, our high-resolution structure of the erastin-bound xCT–4F2hc complex may provide a structural basis for the rational design of potent inducers of ferroptosis, leading to more effective therapeutic strategies.

## Supplementary information

Supplementary information

## Data Availability

Atomic coordinates and EM density maps of the erastin-bound xCT-4F2hc complex (PDB: 7EPZ; EMDB: EMD-31251 for the entire map, and EMD-31252 for the TM-focused refined map) have been deposited in the Protein Data Bank and the Electron Microscopy Data Bank, respectively.
