# Supplementary material for: The structure of erastin-bound xCT–4F2hc complex reveals molecular mechanisms underlying erastin-induced ferroptosis
Source: Cell Res. 2022 Mar 29;32(7):687–90. doi: 10.1038/s41422-022-00642-w (PMC9253326; doi:10.1038/s41422-022-00642-w)
Supplement: Supplementary file 1 — Supplementary information [file 41422_2022_642_MOESM1_ESM.pdf]

Supplementary Information for

The structure of erastin-bound xCT-4F2hc complex reveals molecular mechanisms underlying erastin-induced ferroptosis

Renhong Yan[1#], Enjun Xie[2,3#], Yaning Li[4#], Jin Li[2,3#], Yuanyuan Zhang[1], Ximin Chi[1], Xueping Hu[5], Lei Xu[6], Tingjun Hou[5], Brent R. Stockwell[7], Junxia Min[2*], Qiang Zhou[1*], Fudi Wang[2,3 *]

[1] Westlake Laboratory of Life Sciences and Biomedicine, Key Laboratory of Structural Biology of Zhejiang Province, Institute of Biology, Westlake Institute for Advanced Study, School of Life Sciences, Westlake University, Hangzhou 310024, China

[2] The First Affiliated Hospital, The Fourth Affiliated Hospital, School of Public Health, Institute of Translational Medicine, Cancer Center, State Key Laboratory of Experimental Hematology, Zhejiang University School of Medicine, Hangzhou 310058, China

[3] The First Affiliated Hospital, The Second Affiliated Hospital, Basic Medical Sciences, School of Public Health, Hengyang Medical School, University of South China, Hengyang 421001, China

[4] Beijing Advanced Innovation Center for Structural Biology, Tsinghua-Peking Joint Center for Life Sciences, School of Life Sciences, Tsinghua University, Beijing 100084, China

[5] Hangzhou Institute of Innovative Medicine, College of Pharmaceutical Sciences, Zhejiang University, Hangzhou 310058, China

[6] Institute of Bioinformatics and Medical Engineering, Jiangsu University of Technology, Changzhou 213001, China

[7] Department of Biological Sciences and Department of Chemistry, Columbia University, New York, USA

[#] These authors contributed equally.

*Correspondence to: fwang@zju.edu.cn (F.W.), zhouqiang@westlake.edu.cn (Q.Z.), or junxiamin@zju.edu.cn (J.M.)

Materials and Methods

Data availability

Atomic coordinates and EM density maps of the erastin-bound xCT-4F2hc complex (PDB: 7EPZ; EMDB: EMD-31251 for the entire map, and EMD-31252 for the TM-focused refined map) have been deposited in the Protein Data Bank (http://www.rcsb.org) and the Electron Microscopy Data Bank (https://www.ebi.ac.uk/pdbe/emdb/).

Protein preparation

The full-length human *SLC7A11* cDNA (expressing the xCT protein; accession number: NM_014331.4) was subcloned into the pCAG vector containing an N-terminal FLAG tag, and isoform b of the *SLC3A2* cDNA (expressing the 4F2hc protein; accession number: NM_001012662.2) was subcloned into the pCAG vector containing an N-terminal 10×His tag.

For protein production, xCT and 4F2hc were co-expressed in HEK293F cells (Invitrogen), which were cultured in SMM 293T-I medium (Sino Biological Inc.) at 37°C in 5% CO_2 in a Multitron-Pro incubation shaker (Infors) at 130 rpm.

For transfection, 0.75 mg of the xCT-expressing plasmid and 0.75 mg of the 4F2hc-expressing plasmid were preincubated with 3 mg polyethylenimine (Polysciences) in 50 ml fresh medium. After incubation for 15 min, the mixture was added to a liter of cell culture medium containing approximately 2.0×10^6 cells/ml. After 48 hours, the cells were collected and resuspended in a buffer containing 25 mM Tris (pH 8.0), 150 mM NaCl, and the protease inhibitors aprotinin (1.3 µg/ml, Amresco), pepstatin (0.7 µg/ml, Amresco), and leupeptin (5 µg/ml, Amresco).

For protein purification, the transfected cells were incubated at 4°C for 2 hours in 1% (w/v) LMNG (Anatrace) supplemented with 0.1% (w/v) cholesteryl hemisuccinate Tris salt (Anatrace), and then centrifuged at 18,700×*g* for 45 mins to remove cellular debris. The supernatant was loaded onto an anti-FLAG M2 affinity resin (Sigma). The resin was then rinsed with wash buffer 1 containing 25 mM Tris (pH 8.0), 150 mM NaCl, and 0.04% (w/v) GDN, and then eluted with wash buffer 1 containing 0.2 mg/ml FLAG peptide. The eluate was further purified using Ni-NTA affinity resin (Qiagen). The protein-loaded Ni-NTA resin was then rinsed with wash buffer 2 containing 25 mM

Tris (pH 8.0), 150 mM NaCl, 0.04% (w/v) GDN, and 10 mM imidazole, and the proteins were eluted using wash buffer 2 containing 290 mM imidazole. The resulting protein complex was then subjected to size-exclusion chromatography (Superose 6 Increase 10/300 GL, GE Healthcare) in a buffer containing 25 mM Tris (pH 8.0), 150 mM NaCl, and 0.02% (w/v) GDN. The peak fractions were collected and used for cryo-EM or to measure *in vitro* transport activity.

In vitro transport activity assay

Liposomes and proteoliposomes were prepared as described previously[1], with slight modification. The reaction buffer inside the liposomes and proteoliposomes contained 20 mM potassium phosphate (pH 6.5), 150 mM KCl, and 20 mM L-glutamic acid monosodium salt hydrate (Sigma). All transport activity assays were performed at room temperature. In brief, 100 μl of reaction buffer containing 20 mM potassium phosphate (pH 6.5), 150 mM KCl, and 5 μM (0.1 μCi) L-[^{14}C]-cystine (PerkinElmer Life Sciences) was added to 4 μl of proteoliposomes to initiate substrate uptake. After 10 min, uptake was stopped by rapidly filtering the reaction solution through a 0.22-μm GSTF filter (Millipore), and the filter was washed with 2 ml ice-cold wash buffer containing 20 mM potassium phosphate (pH 6.5) and 150 mM KCl. The filter was then used for liquid scintillation counting.

For the inhibition assay, proteoliposomes were preincubated with 40 μM erastin (Sigma) or 1 mM sulfasalazine (MedChemExpress) for 30 mins. In each measurement, liposomes containing no protein were used as a negative control.

Cryo-EM sample preparation and data acquisition

Samples of the purified xCT-4F2hc complex were concentrated to approximately 10 mg/ml and incubated with 200 μM erastin for 2 hours, after which 3.5-μl aliquots were applied to glow-discharged Quantifoil holey carbon grids (Au R1.2/1.3). The grids were then blotted for either 3.0 s or 3.5 s and then flash-frozen in liquid nitrogen–cooled liquid ethane using a Mark IV Vitrobot (Thermo Fisher Scientific). The prepared grids were transferred to a Krios electron microscopy (Thermo Fisher Scientific) operating at 300 kV and equipped with a Gatan K3 detector and GIF Quantum energy filter, with a 20-eV slit width used during imaging. Movie stacks were automatically collected using AutoEMation as described previously[2], with a defocus range from -1.2 μm to -

2.2 μm in the super-resolution mode at a nominal magnification of 81,000×. Each stack contained 32 frames with an exposure time of 0.08 s per frame and a total dose rate of approximately 50 $e^{-}/(Å^{2})$. The stacks were motion-corrected using MotionCor2 as described previously[3] and binned 2-fold, resulting in a pixel size of 1.087 Å. In addition, dose weighting was performed, and the defocus values were estimated using Gctf [4].

Data processing

Suitable micrographs were manually selected, and particles were automatically selected using Relion 3 as described previously[5-8]. After 2D classification, suitable particles were selected and subjected to a global angular searching 3D classification process using the cryo-EM map of the LAT1-4F2hc complex as the initial model with only one class. For each of the final few iterations of the global angular searching 3D classification process, a local angular searching 3D classification step was performed, during which the particles were classified into four classes. Non-redundant particles were selected from the local angular searching 3D classification, and these selected particles were then subjected to multi-reference 3D classification, local defocus correction, 3D auto-refinement, and post-processing. To further improve the map quality in the transmembrane (TM) region, we performed focused 3D classification and auto-refinement by applying the appropriate mask to the TM region.

The 2D classification, 3D classification, and 3D auto-refinement steps were performed using Relion 3. The resolution was estimated using the gold standard Fourier shell correlation 0.143 criterion[9] with high-resolution noise substitution[10].

Model building and structure refinement

The atomic model of the erastin-bound xCT-4F2hc complex was built based on its cryo-EM map. A Chainsaw model was first generated for the xCT-4F2hc complex using the structure of the LAT1-4F2hc complex (PDB ID: 6IRT) as a template. Further modeling was performed manually in Coot as described previously[11], with aromatic residues as markers, the density of which was clearly identifiable in the cryo-EM map. During model building, each residue was manually checked based on its chemical properties.

Structure real space refinement was performed using Phenix as described previously[12], with secondary structure and geometry restraints designed to prevent structure overfitting. To monitor for overfitting of the model, the model was refined against one of two independent half-maps from the gold standard 3D refinement approach; the refined model was then tested against the other map. Statistics associated with data collection, 3D reconstruction, and model refinement are presented in Table S1.

Generation of stable cell lines

The full-length human *SLC7A11* cDNA was subcloned into the pTSB-CMV-MCS-3FLAG lentivirus vector (TranSheepBio, Shanghai, China) with an N-terminal FLAG tag. The Q191A, F254A, and F336A mutations were introduced using the Fast Mutagenesis kit (Vazyme Biotech, Nanjing, China) in accordance with the manufacturer's instructions. All virus constructs were co-transfected into HEK293T cells together with the pSPAX2.0 packaging plasmid and the pMD2.G envelope plasmid (TranSheepBio) using Hieff Trans™ Liposomal Transfection Reagent (40802ES03, Yeasen, Shanghai, China), and lentivirus-containing supernatants were collected 24, 48, and 72 hrs after transfection and used to infect HeLa and HT-1080 cells (Cell Bank at the Chinese Academy of Sciences, Shanghai, China). We also used p459 plasmid with two sgRNAs to knockout SLC7A11 in HT-1080 cells. The sgRNA1: ACTGCCTCCTTTGGTCTGAA and the sgRNA2: ATCACAGGCTTTATATCCTG. Stably transfected cells were selected using 750 μg/ml (HT-1080 cells) Geneticin sulfate (T6512, TargetMol, Boston, MA, USA) or 5 μg/ml (HeLa cells) puromycin (TargetMol), and overexpression was confirmed using western blot analysis.

Western blot analysis

Total proteins were prepared by homogenizing the cells in RIPA buffer (R0020, Solarbio, Beijing) and protease inhibitors. The supernatant was collected after centrifugation at 4°C, and protein concentration was measured using a BCA Protein Assay Kit (Beyotime Biotech, Shanghai, China). A total of 30-50 μg of denatured proteins was loaded and separated on a 10% SDS polyacrylamide gel; the proteins were then transferred to a PVDF membrane, which was blotted using the following primary antibodies: anti-xCT (12691, Cell Signaling Technology), anti-FLAG (A8592, Sigma),

and β-Actin (AC026, ABclonal). The membranes were then washed and probed with the appropriate horseradish peroxidase (HRP)-conjugated secondary antibodies. The signal was detected using ECL Western Blotting Substrate (32106, Thermo Scientific).

Cell viability assay

Cells were seeded in 96-well plates at a density of 6,000 cells per well overnight, and then treated with various concentrations of erastin (T1765, TargetMol), IKE (T5523, TargetMol) or sulfasalazine (SAS, HY-14655, MedChemExpress, Monmouth Junction, NJ, USA) for the indicated times. For the time-course experiments, HeLa cells were treated with 20 μM erastin for 24, 36, 48, and 72 hrs, while HT-1080 cells were treated with 2 μM erastin or 1μM IKE for 12, 24, and 48 hrs. L-Cystine free DMEM was bought from Gibco (21013-024), and then added 2mM L-Glutamine (25030-081, Gibco) and 0.2mM L-Methionine (M9625, Sigma). At the indicated time points, cell viability was measured using the CCK-8 cell counting kit (MedChemExpress) or the CellTiter-Glo Luminescent Cell Viability Assay (G7572, Promega) in accordance with the respective manufacturer's instructions.

Lipid ROS measurements

HT-1080 and HeLa cells were plated in 12-well plates at a density of 200,000 cells per well. The following day, HT-1080 cells were treated for 6 hrs with 4 μM erastin either with or without 2 μM Fer-1, while the HeLa cells were treated for 24 hrs with 20 μM erastin either with or without 4 μM Fer-1; the cells were then incubated with 5 μM C11-BODIPY 581/591 (Invitrogen Life Technologies) for 30 mins at 37°C. Fluorescence-activated cell sorting (FACS) was performed using a NovoCyte flow cytometer (ACEA Biosciences) or a Cytoflex S (BD Biosciences), and FACS data were analyzed using FlowJo (BD Biosciences).

[^{14}C]-Cystine Uptake Assay

The experiments were performed as previously described[13-15] with some modifications. One-hundred thousand (100,000) HT-1080 cells/well were seeded overnight in 12-well plates. The cells were then pretreated with indicated dose of erastin or DMSO as control for 2 hours and then washed twice in prewarmed Na^+-free uptake buffer (137 mM

choline chloride, 3 mM KCl, 1 mM $CaCl_2$,1 mM $MgCl_2$, 5 mM D-glucose, 0.7 mM K_2HPO_4, and 10 mM HEPES [pH7.4]). After that, cells were incubated for 10 min at 37C in 0.6 ml of uptake buffer and then the buffer was replaced with 0.25 ml uptake buffer containing 0.05 μCi of L-[1,2,1',2'-^{14}C]-cystine (NEC854, Perkin Elmer, USA) with or without indicated dose of erastin for 15 min at 37C. Cells were then washed three times with ice-cold uptake buffer and lysed in 500 μl 0.1 M NaOH. The lysate was added to 10 ml of scintillation cocktail and stored in dark for 12 hours. Radioactive counts were obtained for 5 minutes by using a Tri-CARB 4910TR liquid scintillation counter (Perkin Elmer). All measurements were performed in triplicate for each condition.

GSH and GSSG measurements

Cells were incubated overnight in 6-well plates; when the cells reached 70-80% confluence, they were treated for 24 hrs with 20 μM erastin either with or without 4 μM Fer-1. The cells were then washed with ice-cold phosphate-buffered saline (PBS) and harvested in ice-cold methanol containing 50 mM N-ethylmaleimide. The samples were then injected onto a UPLC BEH C18 column (Waters) at a flow rate of 0.4 ml/min as previously reported[16]. Data were obtained using a 1290 Infinity II UHPLC system (Agilent Technologies) coupled to a 6470A Triple Quadrupole mass spectrometer (Agilent Technologies) and processed using Mass Hunter Workstation Software, version B.08.00 (Agilent Technologies); finally, the GSH/GSSG ratio was calculated.

Molecular docking

The cryo-EM structure of the erastin-bound xCT-4F2hc complex was used for molecular docking. The protein structure was generated using the Protein Preparation Wizard in Schrödinger (Release 2018-1: Maestro; Schrödinger, LLC). The binding site was defined as the region centered at the center-of-mass of erastin with a size of 10 Å × 10 Å × 10 Å using the Receptor Grid Generation component of Glide. The IKE molecule processed using LigPrep was docked into the prepared structure using the Glide module, and the binding energies were scored using the Glide SP scoring mode.

Statistical analysis

All summary data are presented as the mean ± SD. Data were compared using the unpaired Student's *t*-test or one-way ANOVA followed by a Bonferroni post hoc

analysis, where appropriate. Data were analyzed using Prism 8.3.0 (GraphPad), and differences with a p-value <0.05 were considered statistically significant.

Supplementary Table

Table S1. Summary of data collection, processing and model statistics for the erastin-bound xCT-4F2hc complex

**Data collection**	
EM equipment	Titan Krios (Thermo Fisher Scientific)
Voltage (kV)	300
Detector	Gatan K3 Summit
Energy filter	Gatan GIF Quantum, 20 eV slit
Pixel size (Å)	1.087
Electron dose (e-/Å2)	50
Defocus range (μm)	-1.2 ~ -2.2
Sample	xCT
Number of collected micrographs	4,999
Number of selected micrographs	4,028
**3D Reconstruction**	
Software	Relion 3.0
Number of used particles (Overall)	114,524
Resolution (Å)	3.4
Symmetry	C1
Map sharpening B-factor ($Å^2$)	-150
**Refinement**	
Software	Phenix
Cell dimensions	
a=b=c (Å)	278.272
α=β=γ (˚)	90
**Model composition**	
Protein residues	920
Side chains assigned	920
Sugar	8
ligand	2
Phospholipid	2
Erastin	1
**R.M.S. deviations**	
Bonds length (Å)	0.007
Bonds Angle (˚)	1.049
**Ramachandran plot statistics (%)**	
Preferred	90.84
Allowed	9.05
Outliers	0.11

Supplementary Figures

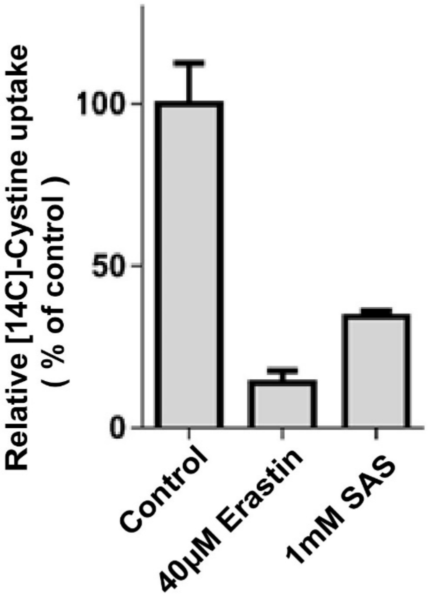

Fig. S1 | Transport assay for the purified xCT-4F2hc complex.

A liposome-based counter-flow assay was performed using the purified wild-type xCT-4F2hc complex. The uptake of [^{14}C]-cystine into the proteoliposomes is shown relative to control; where indicated, the proteoliposomes were preincubated with 40 μM erastin or 1 mM sulfasalazine (SAS) for 30 mins. For each measurement, liposomes containing no proteins were used as a negative control.

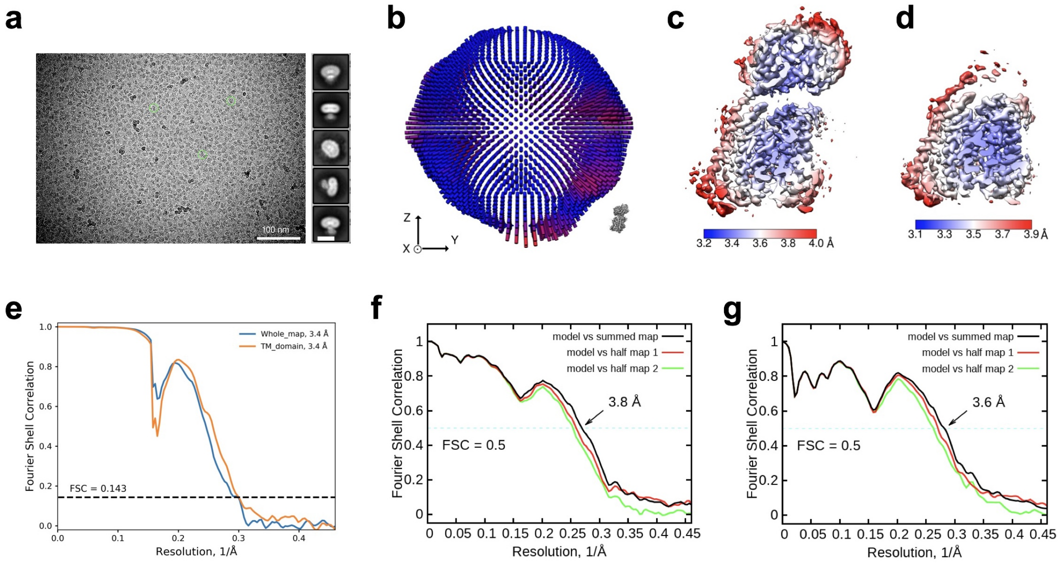

Fig. S2 | Cryo-EM analysis of the erastin-bound xCT-4F2hc complex.

a, Representative cryo-EM micrograph (left) and 2D class averages (right) of cryo-EM particle images. The scale bar in the 2D class averages represents 10 nm. **b,** Euler angle distribution in the final 3D reconstruction. **c-d,** Local resolution map for the 3D reconstruction of the overall structure (**c**) and TM region (**d**) of the complex. **e,** Gold standard Fourier shell correlation (FSC) curve of the overall structure (blue) and TM region (orange) of the complex. **f,** FSC curve of the refined model versus the overall map (black); the model refined against half map 1 versus the overall map is shown in red, and the model refined against half map 1 versus half map 2 is shown in green. The slight difference between the red and green curves indicates that the refinement of the atomic coordinates is not sufficiently overfit. **g,** Same as (**f**), showing the model of the local refined TM region of the erastin-bound xCT-4F2hc complex.

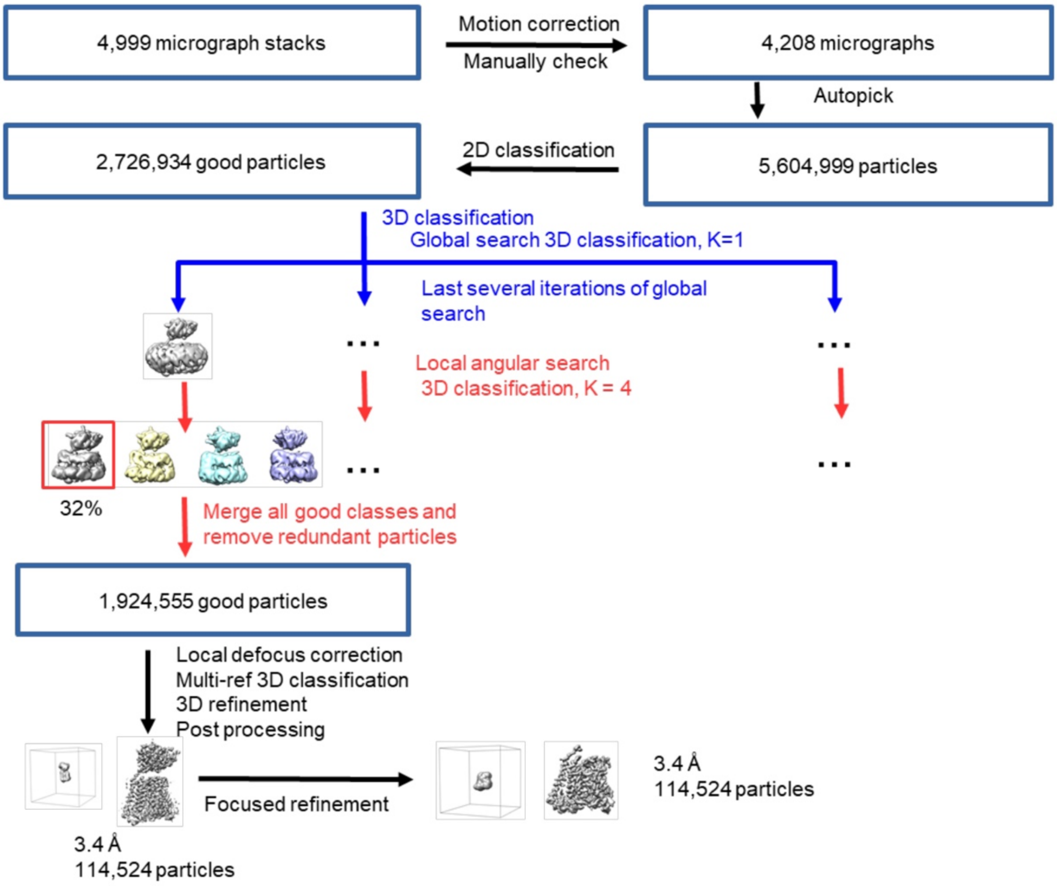

Fig. S3 | Cryo-EM data processing workflow for the erastin-bound xCT-4F2hc complex.

For details, please see the Materials and Methods. “K” refers to the class number of 3D classification.

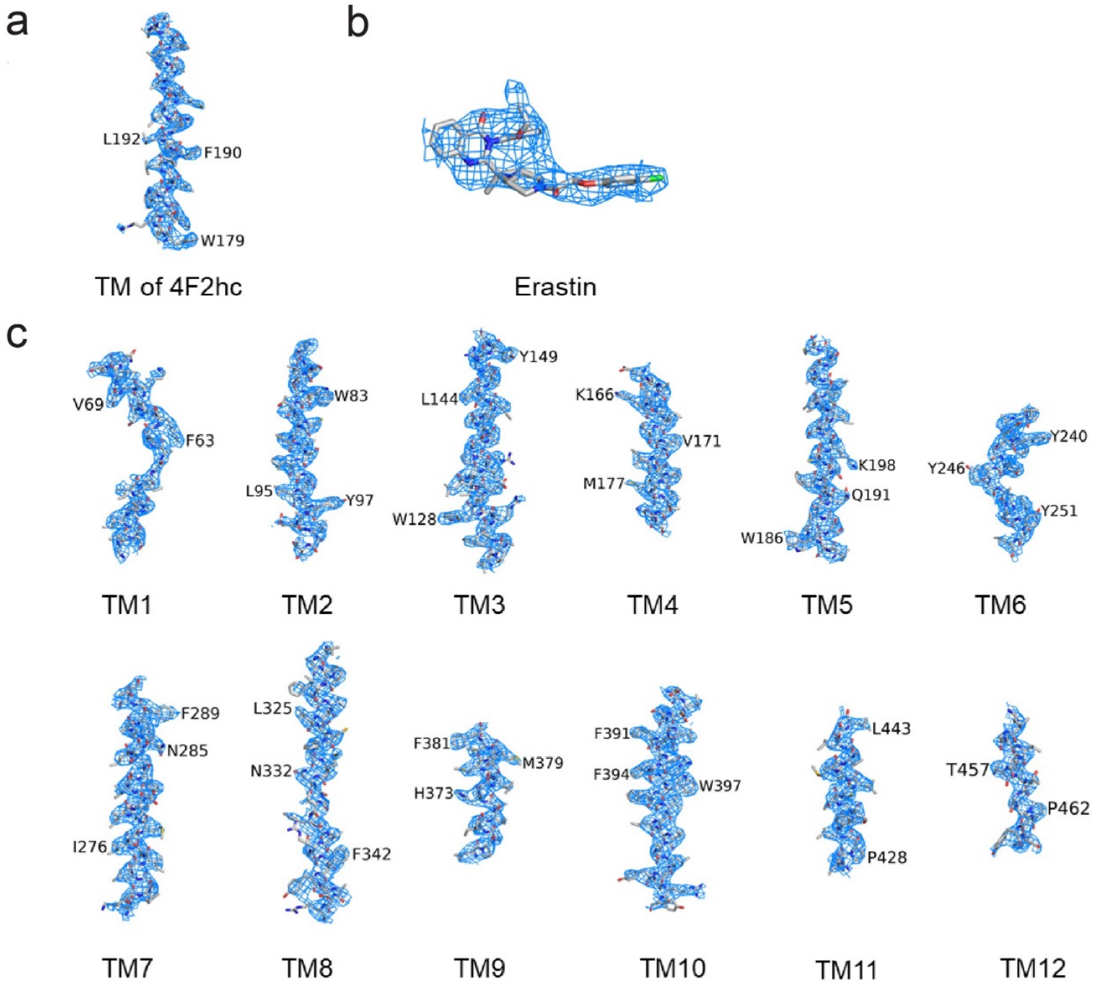

Fig. S4 | Cryo-EM density maps of the indicated transmembrane (TM) regions and the erastin molecule in the erastin-bound xCT-4F2hc complex.

a, Cryo-EM density map of the TM domain in 4F2hc shown at a threshold of 8 σ. **b,** Cryo-EM density map of the erastin molecule shown at a threshold of 6 σ. **c,** Cryo-EM density maps of the 12 TM domains in xCT shown at a threshold of 8 σ.

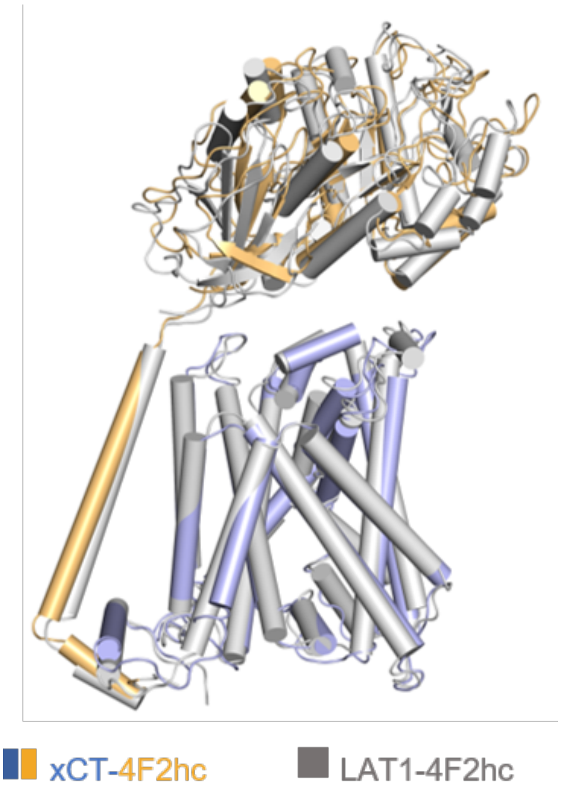

Fig. S5 | Structural comparison between the xCT-4F2hc complex and the LAT1-4F2hc complex.

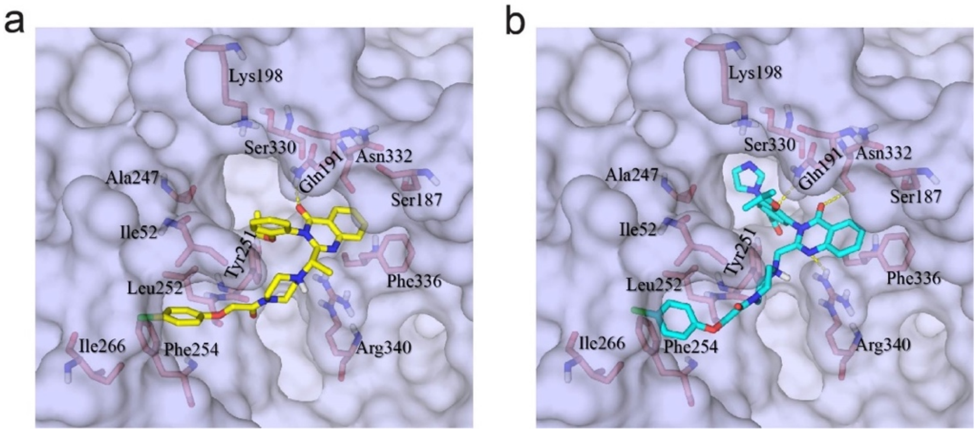

Fig. S6 | Molecular docking of the IKE molecule in the xCT-4F2hc complex.
a, Cryo-EM structure of erastin-bound xCT. **b,** The predicted binding position of imidazole ketone erastin (IKE) in the erastin-binding site. Amino acid residues refer to the xCT protein.

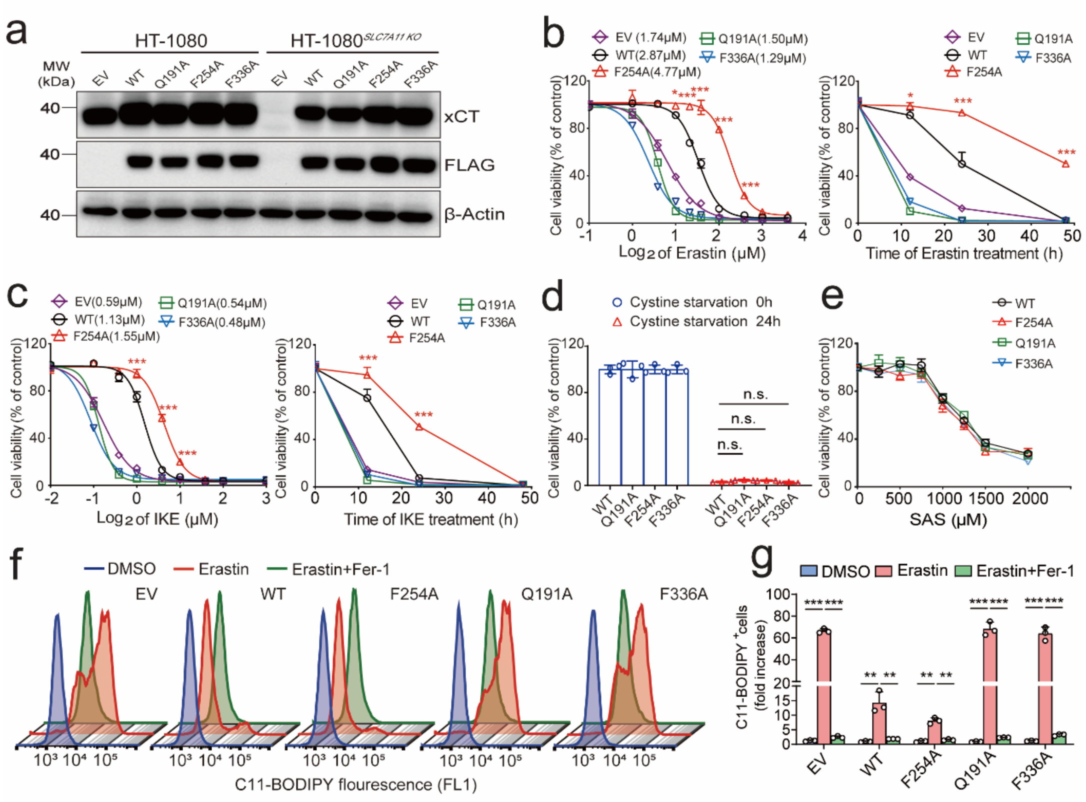

Fig. S7 | The role of xCT WT and mutant forms in erastin or IKE induced ferroptosis in HT-1080 cells and cystine starvation or SAS treatment in xCT KO HT-1080 cells.

a, Western blots of xCT, FLAG, and β-actin protein in HT-1080 and HT-1080 SLC7A11 knockout cells stably expressing an empty vector (EV) or either wild-type (WT) or the indicated mutant forms of FLAG-tagged xCT. **b** Dose-response curve for erastin measured at 12 hrs (left， upper labeled with IC_{50} respectively) and time course measured using 2 μM erastin (right) in HT-1080 cells stably expressing an empty vector (EV), wild-type (WT) xCT, or the indicated mutant forms of xCT. **c** Dose-response curve for IKE measured at 12 hrs (left， upper labeled with IC_{50} respectively) and time course (right) measured using 1 μM IKE in HT-1080 cells stably expressing an empty vector (EV), WT xCT, or the indicated mutant forms of xCT. **d,** xCT KO HT-1080 cells stably expressing wild-type (WT) xCT, or the indicated mutant forms of xCT were seeded overnight, and then replaced with cystine free medium for 24 hrs, cell viability were measured. **e,** Dose-response curve for sulfasalazine (SAS) measured at 24 hrs in xCT KO HT-1080 cells stably expressing wild-type (WT) xCT, or the indicated mutant forms of xCT. **f, g** Erastin-induced lipid ROS production was measured by performing flow cytometry using C11-BODIPY in HT-1080 cells stably expressing an empty vector (EV), WT xCT, or the indicated mutant forms of xCT, 6 hrs after control treatment (DMSO) or treatment with 4 μM erastin or erastin plus Fer-1. Data are represented as mean ±SD, n = 3. *$p<0.05$, **$p<0.01$, ***$p<0.001$.

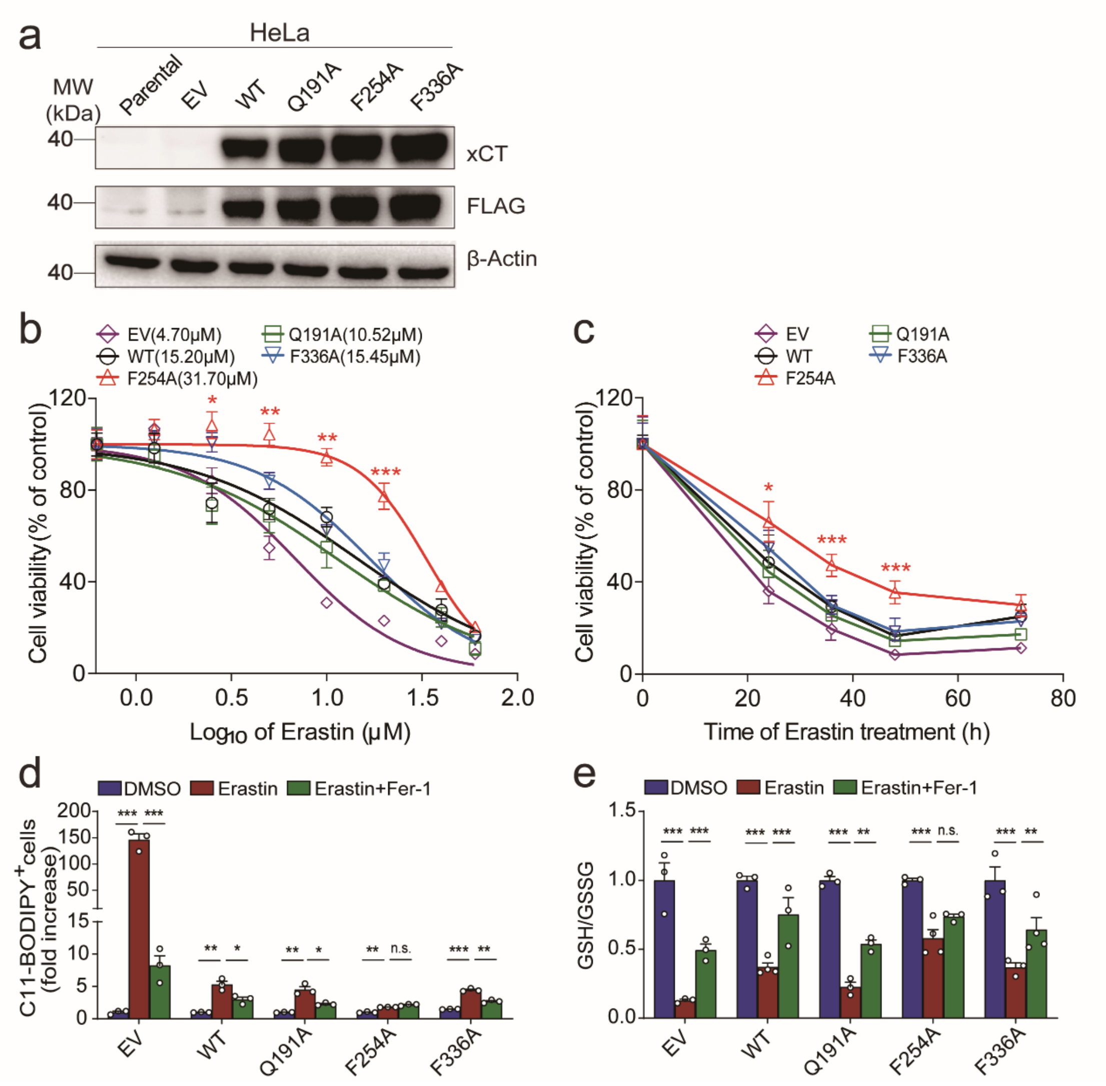

Fig. S8 | Western blot analysis and the results of cell viability, lipid-ROS, GSH/GSSG in HeLa cells.

a, Representative western blots of xCT, FLAG, and β-actin protein in Hela cells stably expressing an empty vector (EV) or either wild-type (WT) or the indicated mutant forms of FLAG-tagged xCT. Also shown is the parent HeLa cell line used to generate the stably expressing cell lines. Dose-response curve for erastin measured at 48 hrs (**b,** upper labeled with IC_{50} respectively) and time course (**c**) measured using 20 μM erastin in HeLa cells stably expressing an empty vector (EV), WT xCT, or the indicated mutant forms of xCT (n=4-6 experiments each). **d,** Quantification of erastin-induced lipid ROS production using C11-BODIPY by flow cytometry in HeLa cells stably expressing an empty vector (EV), WT xCT, or the indicated mutant forms of xCT, 24 hrs after control

treatment (DMSO) or treatment with 20 µM erastin or erastin plus Fer-1 (n=3 each). **e,** Summary of the GSH/GSSG ratio measured using LC-MS in HeLa cells stably expressing an empty vector (EV), WT xCT, or the indicated mutant forms of xCT, 24 hrs after control treatment or treatment with 20 µM erastin or erastin plus Fer-1 (n=3-4 each). Summary data are presented as the mean ± SD, and each dot represents an individual experiment. One-way ANOVA; **p*<0.05, ***p*<0.01, ****p*<0.001, and n.s., not significant (*p*>0.05).

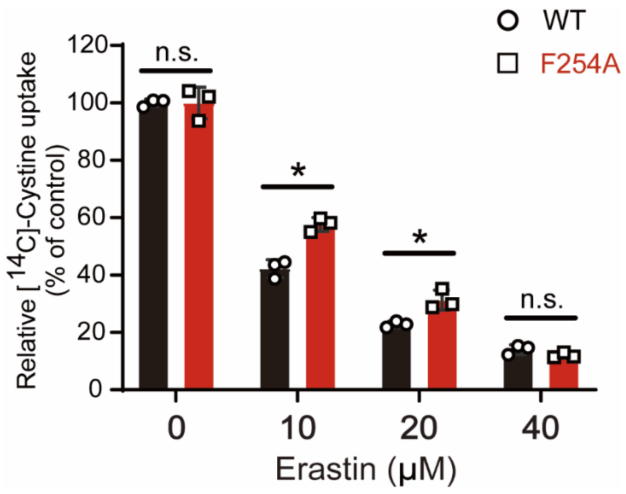

Fig. S9 | Cystine uptake assay for high dose of erastin treatment in xCT KO HT-1080 cells reexpressing either WT or F254A xCT.

Additional Na^+-independent [^{14}C]-cystine uptake assay used 10, 20, 40 µM of erastin in xCT KO cells expressing WT or F254A xCT, which were also pretreated for 2 hours with erastin or DMSO as control. Student's *t*-test, n=3, *$p<0.05$ and n.s., not significant ($p>0.05$).

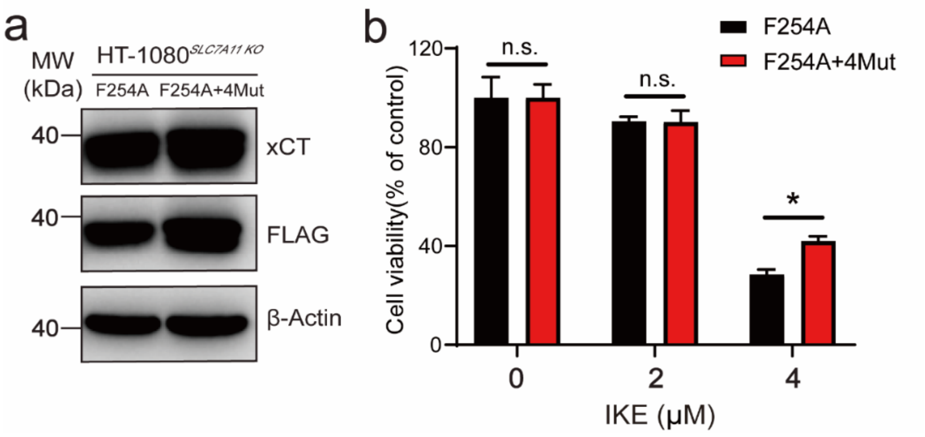

Fig. S10 | Western blot and cell viability analyses of *xCT* knockout HT-1080 cells rexpressing F254A xCT with or without 4 additional xCT mutations (4Mut, Y251A, A247G, K198A, and S330A).

a, Western blots of xCT, FLAG, and β-actin protein in xCT KO HT-1080 cells stably expressing F254A xCT or F254A plus Y251A, A247G, K198A, and S330A mutants xCT (F254A+4Mut). **b**, Cell viability were measured after treatment with indicated dose of IKE at 24 hrs in xCT KO HT-1080 cells stably expressing F254A xCT, or F254A+4Mut xCT. Student's *t*-test, n=3, *$p<0.05$ and n.s., not significant ($p>0.05$).

Supplementary References

1 Yan, R., Zhao, X., Lei, J. & Zhou, Q. Structure of the human LAT1-4F2hc heteromeric amino acid transporter complex. *Nature* **568**, 127-130, doi:10.1038/s41586-019-1011-z (2019).

2 Lei, J. & Frank, J. Automated acquisition of cryo-electron micrographs for single particle reconstruction on an FEI Tecnai electron microscope. *Journal of Structural Biology* **150**, 69-80, doi:10.1016/j.jsb.2005.01.002 (2005).

3 Zheng, S. Q. *et al.* MotionCor2: anisotropic correction of beam-induced motion for improved cryo-electron microscopy. *Nature Methods* **14**, 331-332, doi:10.1038/nmeth.4193 (2017).

4 Zhang, K. Gctf: Real-time CTF determination and correction. *Journal of Structural Biology* **193**, 1-12, doi:10.1016/j.jsb.2015.11.003 (2016).

5 Scheres, S. H. A Bayesian view on cryo-EM structure determination. *Journal of Molecular Biology* **415**, 406-418, doi:10.1016/j.jmb.2011.11.010 (2012).

6 Scheres, S. H. RELION: implementation of a Bayesian approach to cryo-EM structure determination. *Journal of Structural Biology* **180**, 519-530, doi:10.1016/j.jsb.2012.09.006 (2012).

7 Scheres, S. H. Semi-automated selection of cryo-EM particles in RELION-1.3. *Journal of Structural Biology* **189**, 114-122, doi:10.1016/j.jsb.2014.11.010 (2015).

8 Kimanius, D., Forsberg, B. O., Scheres, S. H. & Lindahl, E. Accelerated cryo-EM structure determination with parallelisation using GPUs in RELION-2. *eLife* **5**, doi:10.7554/eLife.18722 (2016).

9 Rosenthal, P. B. & Henderson, R. Optimal determination of particle orientation, absolute hand, and contrast loss in single-particle electron cryomicroscopy. *Journal of Molecular Biology* **333**, 721-745 (2003).

10 Chen, S. *et al.* High-resolution noise substitution to measure overfitting and validate resolution in 3D structure determination by single particle electron cryomicroscopy. *Ultramicroscopy* **135**, 24-35, doi:10.1016/j.ultramic.2013.06.004 (2013).

11 Emsley, P., Lohkamp, B., Scott, W. G. & Cowtan, K. Features and development of Coot. *Acta Crystallographica. Section D, Biological Crystallography* **66**, 486-501, doi:10.1107/S0907444910007493 (2010).

12 Adams, P. D. *et al.* PHENIX: a comprehensive Python-based system for macromolecular structure solution. *Acta Crystallographica. Section D, Biological Crystallography* **66**, 213-221, doi:10.1107/S0907444909052925 (2010).

13 Dixon, S. J. et al. Ferroptosis: an iron-dependent form of nonapoptotic cell death. Cell 149, 1060-1072, doi:10.1016/j.cell.2012.03.042 (2012).

14 Lee, H. et al. Energy-stress-mediated AMPK activation inhibits ferroptosis. Nature cell biology 22, 225-234, doi:10.1038/s41556-020-0461-8 (2020).

15 Hu, K. et al. Suppression of the SLC7A11/glutathione axis causes synthetic lethality in KRAS-mutant lung adenocarcinoma. The Journal of clinical investigation 130, 1752-1766, doi:10.1172/JCI124049 (2020).

16 Fang, X. et al. Loss of Cardiac Ferritin H Facilitates Cardiomyopathy via Slc7a11-Mediated Ferroptosis. *Circulation Research* **127**, 486-501, doi:10.1161/CIRCRESAHA.120.316509 (2020)
